# Spinel-Structured High Entropy Oxides: Low Temperature Synthesis, Characterization, and Potential Applications

**DOI:** 10.1021/acsomega.5c00902

**Published:** 2025-08-25

**Authors:** Irem B. Algan Simsek, Hussein O. Badr, Neal Cardoza, Erika Colin-Ulloa, Gregory R. Schwenk, Kaustubh Sudhakar, Ulf Wiedwald, Michael Farle, Vibha Kalra, Mohamed A. Ibrahim, Lyubov V. Titova, Michel W. Barsoum

**Affiliations:** † Department of Metallurgical and Materials Engineering, 37511 Gazi University, Ankara 06560, Turkey; ‡ Department of Materials Science and Engineering, 6527 Drexel University, Philadelphia, Pennsylvania 19104, United States; § Department of Chemical and Biological Engineering, 6527 Drexel University, Philadelphia, Pennsylvania 19104, United States; ∥ Department of Physics, 8718 Worcester Polytechnic Institute, Worcester, Massachusetts 01609, United States; ⊥ Faculty of Physics and Center for Nanointegration Duisburg-Essen, 27170 University of Duisburg-Essen, Duisburg 47057, Germany; # Robert Frederick Smith School of Chemical and Biomolecular Engineering, 5922 Cornell University, Ithaca, New York 14853, United States

## Abstract

High entropy oxides (HEOs) have recently attracted increasing attention due to their remarkable properties and relatively low cost. Herein we report a simple, highly scalable, and low temperature method for synthesizing spinel (FeNiCoCuZn)_3_O_4_ (HEO). We heated an aqueous solution containing divalent cations in high alkali environments to temperatures of 25 - 95 °C for 24 h under atmospheric pressure. The HEO, synthesized at 95 °C for 24 h in 1 M KOH, was paramagnetic at room temperature, with a magnetic mass susceptibility of χ = (7.5 ± 0.2) × 10^–7^ m^3^·kg^–1^. It demonstrated stable electrochemical lithium storage performance, with a gravimetric capacity of ∼300 mAh·g^–1^ at 100 mA·g^–^^1^. It was also active in the electrocatalytic oxygen evolution reaction with an overpotential of 460 mV in alkaline media. The band gap energies were in the range of 2.4 eV. Our advancement in the synthesis and processing of transition metal-based HEOs will undoubtedly render them a viable solution for next generation materials for energy production and storage.

## Graphical abstract

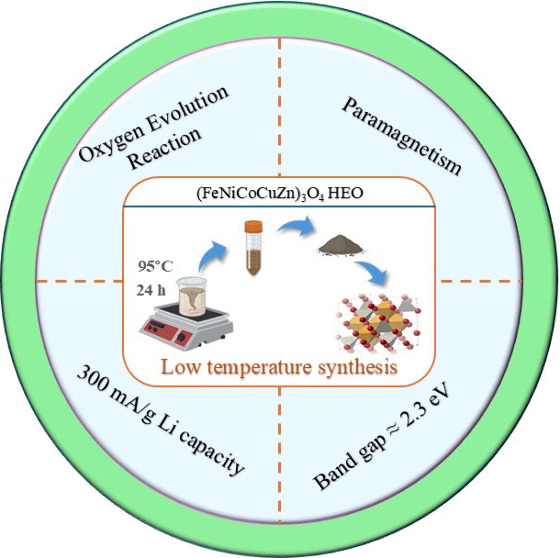

## Introduction

In recent decades, increasing demands for energy and materials have underscored the urgent need to address environmental degradation and energy challenges. Some challenges such as reliance on nonrenewable fossil fuels and the consequent environmental pollution and climate change, along with the growing demand for energy, are pressing concerns that society must address in the next decades. Addressing these issues will not only mitigate the environmental impact of human activities but also shape the future trajectory of the global economy.^1–3^ For instance, the rising demand for portable electronic devices and energy storage has highlighted the need for high-capacity, efficient, and stable battery materials with long cycle lives. Another important issue is the development of clean, highly efficient, and environmentally friendly fuels. Consequently, interest in developing catalysts for hydrogen (HER) and oxygen evolution reactions (OER) has been increasing steadily.^4,5^

Transition metal oxides (TMOs), with a large diversity of electrons in their d orbitals, earth abundance, and relatively low cost, are viable alternatives to platinum group metal electrocatalysts.^6,7^ Similarly, TMOs-based high entropy oxides (HEOs) have garnered a great deal of attention. HEOs feature tunable electronic structures and good catalytic properties, attributed to an abundance of active catalytic sites, making them promising candidates for electrocatalytic,^1–4,8^ photocatalytic,^9^ and energy storage applications.^1,10–12^ HEOs are a class of materials consisting of five or more elements in equal or nearly equal stoichiometric ratios. Their configurational entropy exceeds 1.5*R* (where *R* is the gas universal gas constant), which enhances the solid solution limits of the elements involved.^1,13^

In 2015, Rost et al. first introduced the concept of HEO by reacting equimolar ratios of five different oxides (e.g., MgO, NiO, CoO, CuO, and ZnO) in the 750–1000 °C temperature range to form a single phase with a rock salt structure.^14^ In 2018, Dąbrowa et al. extended the HEO concept to include spinel-type mixed metal oxides.^15^ A single-phase spinel-type (Co,Cr,Fe,Mn,Ni)_3_O_4_ HEO was synthesized for the first time, as confirmed by X-ray diffraction (XRD) and Raman studies. Currently, a crucial strategy in varying the composition of HEOs involves using TMs with similar atomic radii and good mutual solubilities.^16–18^ To date, numerous HEOs with various structures have been reported. However, the spinel-type has garnered particular attention due to its superior magnetic properties, energy storage capabilities, and catalytic performance.^19–22^

HEOs have primarily been synthesized following solid-state reaction pathways, which necessitate high temperatures that can consume significant amounts of energy and time. Alternatively, solvothermal and hydrothermal methods have been employed, involving the use of solvents or aqueous solutions with expensive autoclave systems that are nontrivial to scale up. Controlling the reaction parameters in these methods is challenging, leading to limited commercial applicability.^5,19,21,22^ Certainly, simplicity as well as reduced energy and time consumption are critical considerations in the synthesis of HEO materials, especially for applications such as OER and Li-ion batteries.

Wet chemical synthesis methods, such as sol–gel processes, are increasingly favored due to their advantages in these regards. For instance, Yu et al. synthesized perovskite-type HEOs by a sol-hydrothermal method to benefit from the advantages of both production methods.^23^ Asim et al. synthesized an Al, Mg, Fe, Cu, Ni, and Co-based HEO by a sol–gel autocombustion method followed by a calcination step at 800 °C and obtained a fine crystalline powder.^24^ Similarly, Wang et al. adopted a sol–gel strategy combined with high temperature solid-state calcination, in the 400–900 °C temperature range, and prepared a Co, Mn, Fe, Ni, and Cr-based mesoporous HEO with a spinel structure.^25^ The closest approach to ours for making HEOs is that of Petrovičovà et al., who reacted stoichiometric amounts of 5 select elemental precursors including Mg, Cr, Fe, Mn, Co, Ni, Cu, and Zn acetates and Ti butoxide at 90 °C and calcined the resulting powders in static air at 350 to 900 °C for 2 h.^26^ In all cases, for the wet chemistry-based methods, an additional high temperature annealing treatment to obtain a single-phase HEO structure is required.^3^

Quite recently, we discovered a novel method to prepare metal oxide nanostructures following a one pot, simple, bottom-up approach.^27–40^ The materials made using this new method are named hydroxide-derived nanostructures, or HDNs,^30^ in which we scalably prepare novel metal oxide nanostructures simply by reacting hydroxide aqueous solutions (mostly tetramethylammonium hydroxide, TMAH) with various TM-containing powders at temperatures <100 °C and under ambient pressure. In one case, 12 different commercial and earth-abundant Ti-containing powders such as binary carbides, nitrides, and borides, among others, were converted into new, one-dimensional lepidocrocite (1DL) titanate nanofilaments (NFs).^27–35^ In another, we converted five different Mn-containing powders (Mn_3_O_4_, Mn_2_O_3_, MnB, etc.) into MnO_2_ birnessite-based crystalline 2D flakes.^36–38^ In a third case, reacting hydroxide aqueous solutions of TMAH, KOH, or NaOH with Fe-bearing powders produced Fe_3_O_4_-based ferrite nanoparticles in the 20 nm size range.^39^

The focus of this work is to follow our HDN method to prepare HEOs at temperatures <100 °C and under ambient pressure and to determine their properties. To this effect, we prepared a (FeNiCoCuZn)_3_O_4_ spinel-structured HEO (s-HEO) by reacting transition metal salts with dilute aqueous solutions of NaOH, KOH, and TMAH in the 25–95 °C range for up to 24 h. Based on XRD studies, the resulting HEOs crystallize in the spinel *Fd*3̅*m* (227) structure. Notably, no high temperature annealing steps are needed. We investigated its applications in electrocatalytic OER and as anodes in electrochemical Li-ion batteries. We also report on its magnetic behavior and the UV–vis absorption spectra.

## Results and Discussion

Our synthesis method is shown in Figure 1a. In brief, the TM precursor solutions were prepared by dissolving Fe^2+^, Ni^2+^, Co^2+^, Cu^2+^, and Zn^2+^ water-soluble salts in an equimolar ratio in water to form 0.5 M solutions. In a separate reaction bottle, aqueous solutions of KOH, NaOH, or TMAH were prepared and slowly added to the precursor solution to adjust the solutions’ overall molarity to 0.03, 1, or 7 M. The resulting mixture was then allowed to stir at 25 or 95 °C for up to 24 h. After reaction, the resulting slurry was collected into centrifuge tubes and rinsed with ethanol and water for 4–5 cycles until the pH reached 7. Finally, the sediment was allowed to dry in open air at 50 °C overnight. The nominal resulting HEO composition is (FeNiCoCuZn)_3_O_4_ and will be referred to as “HEO sediment” or s-HEO.

**Figure 1:**
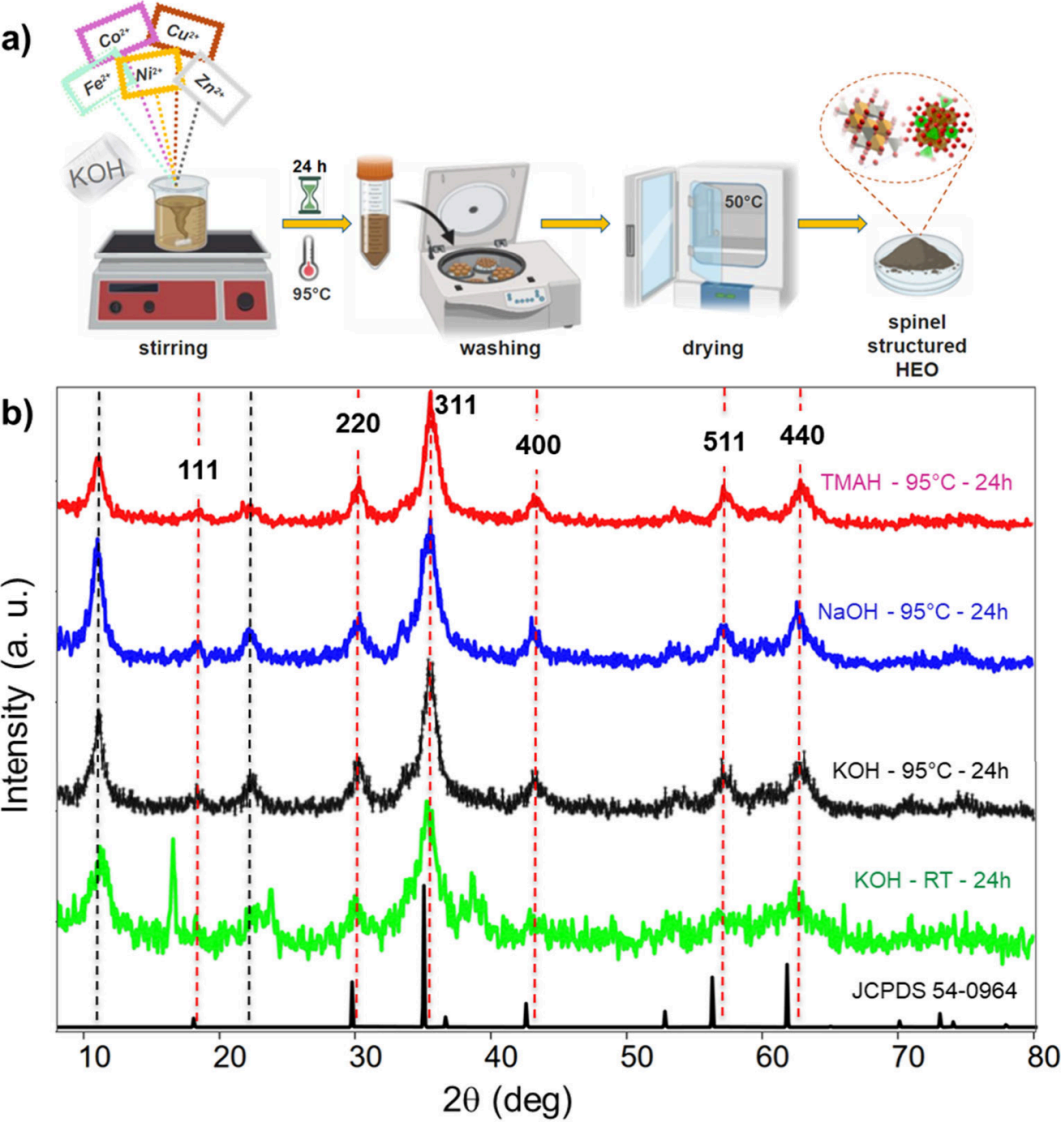
(a) Schematic diagram of HEO preparation procedure. (b) XRD patterns for c-HEO powders prepared by mixing Fe, Ni, Co, Cu, and Zn salt solutions with an aqueous solution of 1 M TMAH at 95 °C (pink), NaOH at 95 °C (blue), KOH at 95 °C (black), and KOH at RT (green). All samples were reacted for 24 h and washed with ethanol and DI water for 4–5 cycles until spontaneous delamination was observed. The resulting colloids were filtered and dried at 50 °C overnight before XRD scanning. The diffraction card JCPDS 54-0964 for the spinel structure is shown in black at the bottom. Vertical dashed red and black lines refer to crystallographic planes of bulk and low-dimensional spinel structures, respectively.

In another set of runs, the resulting powders were further rinsed with DI water until aqueous colloidal suspensions formed spontaneously. The whole suspension was then centrifuged at 3500 rpm for 15 min so that all agglomerates and bulk impurities settled down. The resulting colloidal suspension was collected, filtered via vacuum assisted filtration, and dried at 50 °C overnight before any further characterization. The nominal resulting HEO composition is (FeNiCoCuZn)_3_O_4_ and will be referred to as “HEO colloid” or c-HEO.

### Structural Characterization of HEOs

The crystal structures of the synthesized samples were determined by using powder XRD. Figure 1b plots XRD patterns obtained for c-HEO powder as a function of the treatment reagents and temperatures labeled on the panel. All diffraction patterns shown are quite similar, despite variations in the aqueous reagents used for synthesis (KOH, NaOH, or TMAH) and temperatures (room temperature (RT) vs 95 °C). And while the intensities of the minor peaks vary with the different reagents and temperatures, there are no significant changes in peak positions. Specifically, peaks located at 18.9°, 30.6°, 35.9°, 42.5°, 57°, and 62° 2θ correspond to the (111), (220), (311), (400), (511), and (440) planes of the spinel-phase structure, respectively, which match well with existing literature^3,4,41–44^ and are in agreement with the diffraction card JCPDS 54-0964 for the spinel structure. The remaining low angle peak at 11.1° and its second order located at 22.6° do not match diffraction patterns for any of the constituting metal oxides or hydroxides. To validate this conclusion, we carried out a separate experiment in which aqueous solutions of Fe, Ni, and Co salts were treated in 1 M KOH solution at 95 °C for 24 h. XRD patterns of the resulting powders showed a mixture of Fe_3_O_4_ magnetite along with Co and Ni hydroxides, as can be seen in Figure S1. This led us to the conclusion that the two peaks observed at 11.1° and 22.6° could be attributed to the presence of low-dimensional metal oxide octahedral layers similar to that previously observed by Li et al.^16^

In separate runs, we optimized the experimental conditions to prepare crystalline (FeNiCoCuZn)_3_O_4_ HEOs. In these runs, XRD patterns were collected from s-HEO samples (i.e., from the resulting sediments and not just the filtered colloids). Figure S2a–c shows the effect of KOH molarity, reaction time, and temperature on the XRD patterns of the s-HEO samples, respectively. In Figure S2a, HEOs that were processed with 0.03 M KOH at RT exhibit no crystallinity, while those processed with 1 M KOH developed more prominent crystallinity. At 95 °C, s-HEO samples prepared using 1 and 7 M KOH solutions (Figure S2b,c) have the same structures and crystallinity for the most part. Note that the peak intensity and thus the phase crystallinity increased as the reaction time increased from 2 to 24 h and as the reaction temperature increased from RT to 95 °C (see Figure S2b,c). As a result, henceforth, the HEO synthesis conditions of 1 M KOH, 95 °C, and 24 h were selected as the optimum synthesis conditions for further characterization.

Notably all XRD reflections of c-HEO correspond to the spinel-phase structure (Figure 1b). Conversely, all XRD patterns of s-HEO showed an additional peak at 38.7° that most probably corresponds to copper oxide impurities.^45,46^ Therefore, c-HEO samples were used for further characterization unless mentioned otherwise.

The calculated lattice parameters of our spinel HEOs are *a* = *b* = *c* = 8.337 Å, and their space group is spinel *Fd*3̅*m*. The average crystallite sizes, calculated from the XRD patterns, using the Scherrer formula, are summarized in Table S2 and show that after 24 h reaction times, the average crystallite size is ∼11 nm for the c-HEO samples. In general, these values are smaller than similar studies in the literature.^3,4,41,42,44^ The average crystallite size of the RT run, at 4 nm, is quite small (not shown).

### Morphology and Elemental Mapping of HEOs

A typical scanning electron microscope (SEM) micrograph of the HEO colloid samples reacted in 1 M KOH solution at 95 °C for 1 day are shown in Figure 2a, where agglomerated particles are clearly observed. These are composed of smaller nanoparticles, as revealed by the transmission electron microscope (TEM) micrograph shown in the inset in Figure 2a. Based on the SEM micrographs of other c-HEO samples reacted under different conditions shown in Figure S3, we reach the same conclusion. Since the sizes of all particles observed were in the 10–50 μm range, we conclude that the primary HEO particles have a tendency to self-agglomerate.

**Figure 2:**
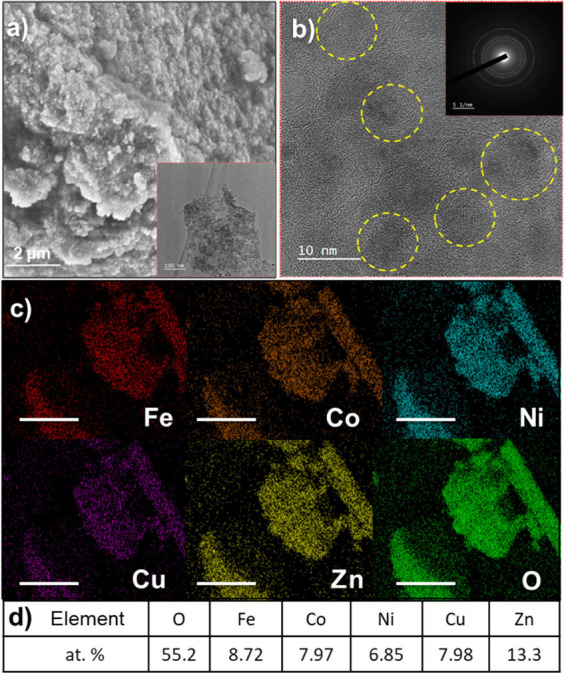
Characterization of c-HEO prepared using 1 M KOH at 95 °C for 24 h. (a) Typical SEM micrograph showing an HEO agglomerate in the micrometer range. Inset is a low magnification TEM micrograph showing the length, stacking, and dispersion of an HEO particle. (b) High magnification TEM imaging of particle shown in inset in (a) revealing grain boundaries and structural defects (lattice distortions and discontinuous lattice fringes). Inset reveals the SAD pattern collected. Dashed yellow circles highlight crystalline HEO nanoparticles. (c) Elemental distribution of HEO colloid sample. Scale bar shown is 5 μm. (d) Elemental composition of the HEO particle shown in (c).

More importantly, from the energy dispersive X-ray spectroscopy (EDS) maps in an SEM of a typical c-HEO particle shown in Figure 2c, we conclude that Fe, Ni, Co, Cu, Zn, and O are uniformly distributed, on the micrometer scale, with no apparent segregation. Note that the Zn content is slightly higher than that of the other cations. Other studies in the literature have reported that depending on the solubility of the precursor metal salts and their reactions with the solvent, they may show slight deviations from the target composition.^3,5,13,41^ In addition, the uniform distribution of HEOs in this study is attributed to a high entropy stabilization effect.^20^ Figure 2b shows a TEM micrograph of c-HEO, in which crystalline nanoparticles in the 10 nm range can be clearly observed (bounded by dashed yellow circles). Additionally, some structural defects like lattice distortions and discontinuous lattice fringes can also be observed possibly due to the different atomic sizes.^21,47^ Crucially, the selected area diffraction (SAD) pattern (inset in Figure 2b) reveals the presence of three rings corresponding to the (111), (311), and (400) planes of spinel HEOs, respectively, which indicates a polycrystalline structure.^16^

### Magnetic Properties of HEOs

The effects of reaction conditions on the magnetic properties of our HEO samples, measured using a vibrating sample magnetometer (VSM) at 5 and 300 K, are shown in Figure 3a,b, respectively. At 300 K, the response is paramagnetic (Figure 3b) and independent of the reaction conditions. The magnetic susceptibility, χ, for samples reacted at 95 °C is (7.5 ± 0.2) × 10^–7^ m^3^·kg^–1^. At 6.9 × 10^–7^ m^3^·kg^–1^, χ was 10% smaller for samples synthesized at RT.

**Figure 3:**
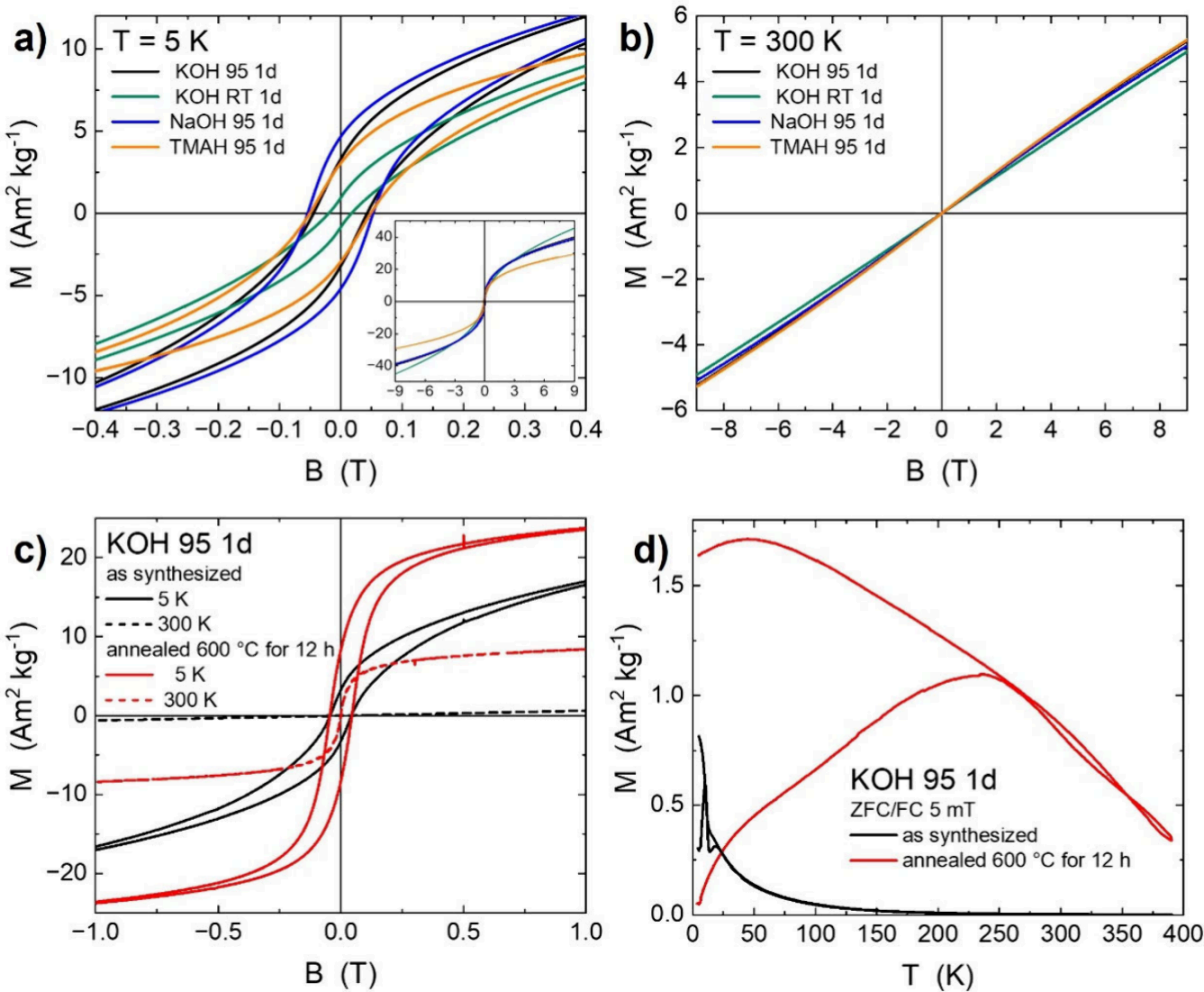
Magnetometry of HEO synthesized with 1 M NaOH at 95 °C (blue), 1 M TMAH at 95 °C (yellow), 1 M KOH at 95 °C (black), and 1 M KOH at RT (green) all for 24 h. (a) Hysteresis loops at *T* = 5 K. Inset shows hysteresis curves up to *B* = ±9 T. (b) Paramagnetic response at 300 K. (c) Hysteresis loops at 5 and 300 K of HEOs synthesized with KOH at 95 °C for 24 h (black) and after annealing at 600 °C for 12 h in ambient air (red). (d) Zero field-cooling/field-cooling in *B* = 5 mT before and after annealing. Labels and curves are color coordinated.

At 5 K, magnetic hysteresis loops were measured with a coercive field μ_0_ *H*_C_ = 50 ± 5 mT for all samples synthesized at 95 °C for 1 day independent of the alkaline reagent used. The inset in Figure 3a presents the data up to ±9 T, showing a nonsaturating behavior in large external fields. Nonetheless, a remarkable magnetization (*M*) of 30–40 Am^2^·kg^–1^ is reached at *B* = 9 T, which is roughly half the value obtained for pure spinel-type ferrimagnetic Fe_3_O_4_ (57 Am^2^·kg^–1^ for 6 nm and 90 Am^2^·kg^–1^ for 12 nm diameter particles).^48,49^ The synthesis of our HEO nanoparticles in KOH at RT, however, shows a less developed hysteresis but larger magnetization values at *B* = 9 T. This we ascribe to the significantly smaller grain/domain size. It is also quite likely that the spinel crystal structure is not well developed for this sample.

The Néel temperature, *T*_N_, or ordering temperature of the sample set can be grouped along the synthesis temperatures.^50,51^ We obtain *T*_N_ = 22 K for samples synthesized at 95 °C and *T*_N_ = 16 K for others prepared at ambient temperature (not shown). These observations motivated us to heat the samples reacted in 1 M KOH at 95 °C for 24 h to 600 °C for 12 h. We expect that such an annealing step drives the HEO sample toward its crystallographic ground state by removing structural defects and increasing the grain size. Interestingly, we do not observe any structural changes, and the crystallite size is similar (within the resolution of our equipment) between the as-synthesized state and the one after annealing at 600 °C for 12 h (see XRD patterns in Figure S5). Magnetometry, on the other hand, is much more sensitive, and subtle variations in magnetization and ordering temperatures are observed. Figure 3c compares the effect of annealing on magnetic behavior. The magnetization *M* = 23.6 Am^2^·kg^–1^ rises by about 40% from *B* = 0 T to *B* = 1 T after annealing at significantly enlarged magnetic susceptibility, as seen by the steeper rise at small fields. Interestingly, the coercive field is constant upon annealing, suggesting that no phase change took place. At *T* = 300 K, the initially paramagnetic response (Figure 1b) changed to a superparamagnetic one with *M* = 8.3 Am^2^·kg^–1^ after annealing.

This becomes clearer in the zero field-cooled/field-cooled (ZFC/FC) experiment at *B* = 5 mT, shown in Figure 3d. The sharply decreasing paramagnetic signal above *T*_N_ = 22 K with practically zero magnetization at *T* = 300 K changes to the typical response of small particles with ordering temperatures well above the measurement interval (3–390 K). Due to the small particle sizes, thermal fluctuations lead to magnetization reversal above the so-called blocking temperature.^18^ The broad peak in the ZFC (lower) curve indicates a broad distribution of the product of particle volumes *V* and effective magnetic anisotropy energy densities *K*_eff_ that can be explained by possible stoichiometric variations on the few-nanometer scale and the large size distribution. This is reflected in a broad distribution of local ordering temperatures, as can be seen in Figure S4. The temperature-dependent magnetization in *B* = 1 T does not obey the typical curve, i.e., a decay according to mean-field theory, but rather reflects a broad distribution of ordering temperatures over several 100 K. This suggests that the magnetic ground state of our spinel nanoparticles is ferrimagnetic for a wide stoichiometry range, which results in strongly varying Néel temperatures (50–500 K) depending on local compositions. To our knowledge, this is the first magnetic study of HEOs synthesized with the (FeNiCoCuZn)_3_O_4_ composition and offers a useful observation on the magnetic complexity and functioning of HEO systems.

### Electrochemical Energy Storage of HEOs

Galvanostatic and potentiostatic tests of our HEOs were run in half cells to understand how they electrochemically behave as anodes in Li^+^-ion cells. The results are shown in Figure 4. Figure 4a shows typical cyclic voltammetry (CV) plots acquired at 0.05 mV·s^–1^ for the first and second cycles. In the first cycle, three reduction (cathodic) peaks in blue are observed. The first peak, at around 0.5 V, is associated with irreversible reduction reaction and the formation of the solid electrolyte interface (SEI) because of the decomposition of the electrolyte.^13,26,47,52^ This irreversible reaction gives rise to shifts of the peaks in the subsequent - orange - cycle at the same scan rate in Figure 4a.^27^ Here, separated and weakened second and third peaks indicate that the activation process is over and the electrode polarization has decreased.^47^

**Figure 4:**
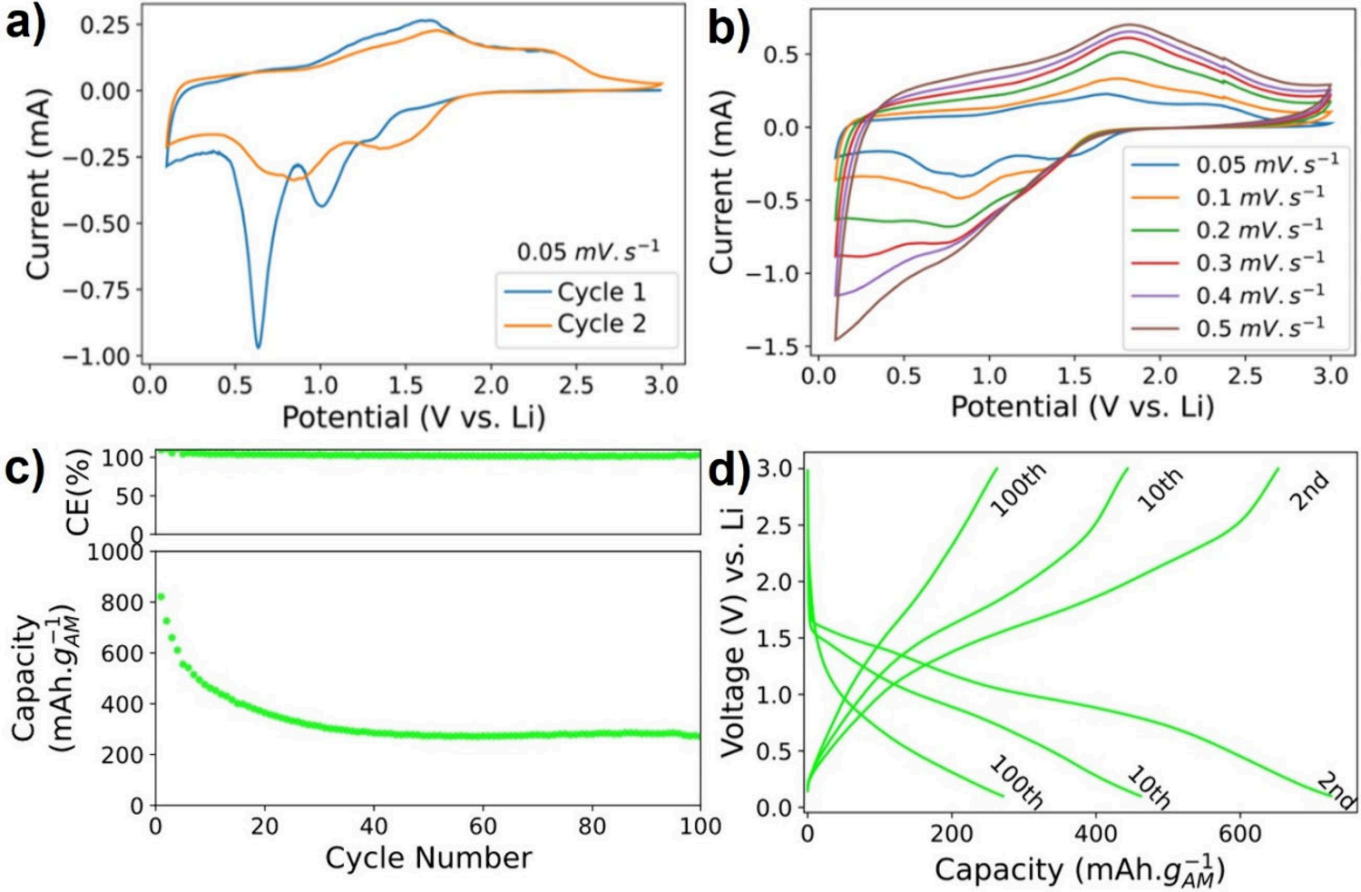
Electrochemical performance of HEO synthesized with 1 M KOH at 95 °C for 24 h. Cyclic voltammetry (a) of the first two cycles, which have three peaks, and (b) peak changes for the reduction and oxidation peaks with varying scan rate. Galvanostatic cycling of HEO anodes with a loading of 2 mg·cm^–2^ and (c) capacity and efficiency data cycled at 100 mA·g^–1^ with stable capacity at 300 mAh·g^–1^. (d) Voltage profile from select cycles showing evolution of peaks; cycle 2 was obtained at a scan rate of 25 mA·g^–1^ and cycles 10 and 100 at 100 mA·g^–1^.

Figure 4b shows CV plots of HEOs as a function of scan rates from 0.05 to 0.5 mV·s^–1^. Here the shifts in peak positions and shapes can be attributed to the changes in the oxidation states of presumably Fe, Co, and Ni. This structural irreversibility can also be seen in the galvanostatic tests of HEOs tested as anodes.^41,53^ The cycling performance, run at 100 mA·g^–1^ and a loading of 2 mg·cm^–2^ , and voltage profiles of our HEO powders are plotted in Figure 4c,d. Plateaus can be seen at around 0.9 and 1.5 V in cycle 2 that are compatible with the CV curves. These two plateaus represent the SEI formation and reaction between HEOs and Li. Furthermore, the lower plateau diminishes between cycle 2 and cycle 10, which indicates the intercalation of Li in the structure is limited at higher C rates possibly because of the incomplete delithiation and pulverization of the powders during cycling.^13^ This incomplete delithiation and pulverization is further exacerbated at sustained higher C rates between cycles 10 and 100, seen as the loss of the upper plateau (Figure 4d). This phenomenon results in a stable final gravimetric capacity of 300 mAh·g^–1^ at 100 mA·g^–1^ (Figure 4c) for 100 cycles.

### Band Gap Measurement of HEOs

Ultraviolet and visible (UV–vis) spectroscopy was utilized to determine the optical absorption of various HEO powders made by filtering colloidal suspensions. Figure 5 shows the Tauc plots, assuming the HEOs are direct band semiconductors. As seen from Figure 5, all HEOs exhibited broad absorption covering the entire visible range, consistent with findings for other spinel structures.^39,54–56^ The results (Figure 5) indicate that the band gap energy, *E*_g_, values ranged from 2.2 to 2.5 eV, which are in the visible range and thus advantageous for photocatalytic and solar cell applications. Given the narrow range of *E*_g_ values, we tentatively conclude that processing conditions play a small, or no, role in determining *E*_g_.

**Figure 5:**
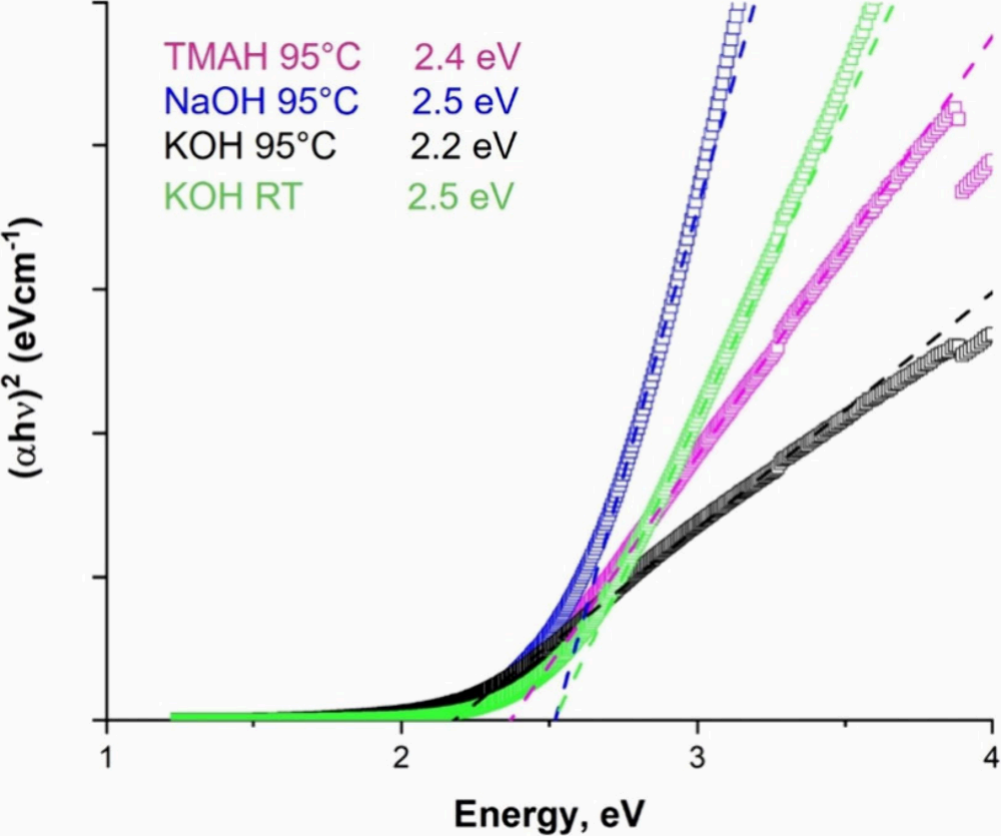
Tauc plots of UV–vis absorption spectra of our HEOs synthesized with NaOH (blue) and TMAH (pink) at 95 °C as well as KOH at RT (green) and 95 °C (black) for 24 h plotted as (α*hv*)^2^ (α: absorption coefficient, *h*: Planck’s constant, ν: light frequency) vs. light energy. Labels and curves are color coordinated.

### Electrocatalytic Performance of HEOs for OER

The OER activity of our HEOs was revealed using a standard three-electrode setup with a 1 M KOH solution. Figure 6 plots a linear sweep voltammetry (LSV) curve showing an onset potential of 1.5 V vs RHE. As reported in similar studies, the catalytic activity of HEOs is driven influentially by composition; for instance, Cu and Zn elements have a positive impact on OER behavior.^4,17,57^ The overpotentials of HEOs were calculated by LSV and are 460 mV (1.69 V against a Ag/AgCl reference electrode) for our HEOs at a current density of 10 mA·cm^–2^. Similar compositions in previous studies are given in Table S3.^4,18,42,58–60^ As seen from here, the number and diversity of components, even if only an element, or the synthesis method changes the synergistic effect in multicomponent systems. For instance, Li et al. used a solvothermal method to synthesize FeNiCoCrMn HEO, while Talluri et al. used reverse coprecipitation, and each group reported dissimilar OER performances.^18,61^ On the other hand, we used a low temperature synthesis method for the same composition but observed different results. All of these results clearly show that more systematic studies are needed to gain an understanding of the underlying reasons in the nature of HEOs.^20,42^

**Figure 6:**
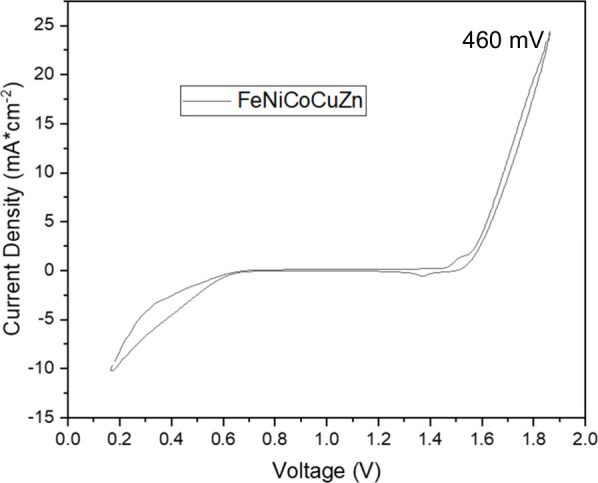
Electrochemical OER activity of our HEO synthesized with 1 M KOH at 95 °C for 24 h. 0.1 M KOH was used as electrolyte solution, and a rotating disk electrode set to 1600 rpm was employed. All voltages were obtained against a Ag/AgCl reference electrode and were converted to compare against the RHE. Polarization LSV curves and overpotentials at a current density are given.

## Conclusions

We synthesized spinel-structured earth-abundant TM-based HEOs at temperatures <100 °C under atmospheric pressure. Characterization results of this study show that all HEO samples synthesized with different alkaline solutions at 95 °C exhibit the same XRD patterns, morphologies, and similar E_g_ values. Thus, it was concluded that KOH is a more suitable option among the other solutions in terms of cost, performance, and environmental health for the synthesis of a spinel (FeNiCoCuZn)_3_O_4_ HEOs. Our results demonstrate that our HEOs exhibit favorable structural, magnetic, electrochemical, and OER characteristics. Notably, a reasonable and simple heat treatment transformed the paramagnetic property into a superparamagnetic one without a phase change. Furthermore, our HEO performed excellently as an anode material for Li-ion batteries. At 2.4 eV, E_g_ is small enough to allow for solar-based applications.

## Experimental Procedures

### Materials Synthesis and Processing

#### Chemicals

Copper­(II) chloride anhydrous (99%, Acros Organics), cobalt­(II) chloride anhydrous (99.7%, Alfa Aesar), nickel­(II) chloride anhydrous (98%, Alfa Aesar), zinc chloride anhydrous (98+%, Alfa Aesar), and iron­(II) sulfate heptahydrate (98%, Alfa Aesar) were used as precursors. Potassium hydroxide (KOH, meets NF/FCC, Fisher Chemical), sodium hydroxide (NaOH, pellets, Fisher Chemical), and tetramethylammonium hydroxide (TMAH, 25 wt % in water, 99.99%, Alfa Aesar) were used as reagents. Ethanol (Pure 200 proof, DECON Lab Inc.) and deionized (DI) water (Millipore, 18.2 MΩ, TOC < 3 ppb) were used for washing. All of the chemicals were used as purchased.

#### Sample Preparation of HEO

Equimolar precursor powders were dispersed in 50 mL of DI water and stirred with a magnetic stir bar for 15 min. Next, 25 mL of a solution of 1 M KOH was slowly added under continuous stirring to adjust the overall molarity of the metal salt solution to 1 M. The mixed salt solutions were allowed to stir at 25 or 95 °C for 24 h. After the reaction, the resulting dark brown solution was collected and washed with ethanol and DI water for 4–5 cycles until spontaneous delamination was observed. The latter colloidal suspension was sonicated using a bath sonicator for 1 h and centrifuged at 3500 rpm for 20 min. The supernatant was then filtered and allowed to dry at 50 °C overnight before any characterization.

The same procedure was used for preparing HEOs using KOH, NaOH, and TMAH solutions of various concentrations (0.03, 1, and 7 M) and with various reaction times (2 h, 24 h, or 7 days). In the TMAH case, 25 mL of the TMAH stock solution (25 wt % corresponding to ∼2.8 M) was slowly added to the metal salts solution to adjust the overall molarity to ∼1 M. All synthesis parameters are given in Table S1.

### X-ray Diffraction (XRD)

A diffractometer (Rigaku Smart Lab XRD, Tokyo, Japan) operated with Cu K_α_ radiation (40 kV and 15 mA) was used to obtain XRD patterns. The powders were scanned in the 2–80° 2θ range with a step size of 0.02° and a dwell time of 1 s. Unless otherwise noted, all powders were dried overnight at 50 °C in open air before the XRD scans.

### Scanning Electron Microscopy

A scanning electron microscope (SEM) (Zeiss Supra 50 VP, Carl Zeiss SMT AG, Oberkochen, Germany), was used to obtain micrographs of our materials. The SEM settings were set to an in-lens detector, a 30 mm aperture, and an accelerating voltage of 3–5 kV. Samples were sputter-coated with platinum. Energy dispersive X-ray spectroscopy (EDS) maps were taken on a spectrometer (Oxford UltiMax 40 mm EDS) with a detector working distance of 15 mm and an accelerating voltage of 20 keV.

### Transmission Electron Microscopy

Transmission electron microscopy (TEM) images accompanied by selected area diffraction (SAD) patterns were acquired on a TEM (JEOL 2100F, Tokyo, Japan) equipped with a Schottky field emission electron source.

### Magnetism

Vibrating sample magnetometry (VSM) was conducted with a physical property measurement system (PPMS) DynaCool in fields of ±9 T and variable temperatures between 5 and 700 K. About 20 mg of the powder was weighed and placed inside synthetic capsules for *T* = 5–390 K. All data have been normalized to the sample masses. At elevated temperatures, the sample powder was mixed with Zircar cement, pasted, and dried onto a heating holder in air. High temperature *M* vs *T* curves were obtained in the temperature range 300–700 K. Since the exact amount of powder cannot be precisely determined, the high temperature signal was scaled to the low temperature magnetization in the interval of 300–400 K by a single factor.

### Electrochemical Testing

HEOs were used as is from the synthesis section as the active material in the battery electrode. The active material was mixed with carbon black (Alfa Aesar, USA, 99%), and polyvinylidene fluoride (MTI, China) in a ratio of 70:20:10, respectively, with *N*-methyl-2-pyrrolidone (Alfa Aesar, USA, 99.5%). These components were mixed in a polypropylene cup with 2 zirconia oxide balls (uxcell, China, 5 mm) in a planetary mixer (Thinky, Japan) for a total of 5 min at 2000 rpm. The resulting slurry mix was then cast on copper (Cu) foil (0.017 mm, MTI China) with a doctor blade set to 0.5 mm and drawn with an automatic blade caster (MTI, China). The wet cast electrode of HEO on Cu was then put in a vacuum oven (TMAXCN, China), heated to 80 °C under vacuum, and cooled back down to room temperature under vacuum. Electrodes 11 mm in diameter were punched from the dried cast electrode with a hole punch of equivalent diameter. The resulting 11 mm electrodes were then used as is in the electrochemical testing.

Electrochemical testing of the HEO electrode was performed in the coin cell format of CR2032. The electrodes were first transferred to an argon glovebox (MBraun, Germany, O_2_ < 1 ppm and H_2_O < 1 ppm) and then assembled in the typical fashion using 2032 cell components (MTI, China), Celgard 2325 (Celgard,USA), and 30 μL of liquid electrolyte LP40 (1 M lithium hexafluorophosphate in a 1:1 (v:v) of ethylene carbonate to dimethyl carbonate) standard composition from Gotion, USA. The cells were half cells with a lithium metal counter and pseudo-reference (MTI, China). The cells were sealed in a coin cell crimper from MTI. These cells were used as is after being rested for 12 h for electrochemical testing. The cyclic voltammetry test was conducted at 0.05–0.5 mV·s^–1^ on a VMP3 potentiostat (Biologic, France). The galvanostatic tests were conducted on a Neware BTS 4000 instrument with 1 C = 1000 mAh·g^–1^. All electrochemical tests were performed in the voltage range of 0.01–3 V vs Li/Li^+^.

### UV–vis

UV–vis spectra of thin films of HEOs deposited on quartz substrates were measured in an integrating sphere in diffuse transmittance geometry using a PerkinElmer Lambda 950 UV–vis/NIR spectrophotometer. The film thicknesses were measured with a Dektak 150 Stylus Profilometer. The band gap energy of HEOs was calculated using the Tauc plot method for direct band gap, (α*hν*)^2^, where α is the absorption coefficient, *h* the Planck constant, and ν the photon frequency.

### Electrocatalytic Oxygen Evaluation Reaction (OER) Testing

Oxygen evolution reaction (OER) electrochemical testing was carried out with the use of a potentiostat (Metrohm Autolab 302N, Utrecht, Netherlands) and a fluorinated ethylene propylene (FEP) cell containing a 0.1 M KOH (from pellets 99.99%, metal basis, Sigma) electrolyte solution in which the HEO film was submerged. Prior to use, the FEP cell was treated by successive boiling and washing with Millipore water (18.2 MΩ·cm, <3 ppb total organic content). Catalyst inks of each HEO material were generated by suspending 4.5 mg of the HEO in 2 mL of IPA along with 4.5 mg of carbon black (Vulcan XC-72R) and 5 μL of an alcohol-based 5 wt % Nafion dispersion (Ion Power). The resulting mixtures were bath sonicated for ∼25 min to form a stable ink. Approximately 8 μL of the resulting catalyst ink was drop-cast onto the surface of an ∼0.196 cm^2^ glassy carbon substrate disk (Sigradur G HTW). Once dried, the ink-coated substrate was inserted into the FEP cell, where it was submerged in the electrolyte solution for OER testing. The potentiostat produced polarization curves across a voltage range of −0.8 to 0.9 V. Additionally, a rotating disk electrode (RDE) (Pine Instruments) set to 1600 rpm was employed during electrochemical testing to ensure that any bubble/solution interfaces were minimized. All voltage results were obtained against a Ag/AgCl reference electrode and were subsequently converted to compare against the reversible hydrogen electrode (RHE). The resulting raw output current values were converted to reflect the current density (mA·cm^–2^).

## Supplementary Material



## Data Availability

The data supporting this article have been included as part of the Supporting Information. For any further data or information, please contact the corresponding authors.

## Notes

Tables detailing experimental parameters and calculated crystallite sizes, figures of additional XRD analysis, additional SEM micrographs, and additional temperature-dependent magnetization graphic (PDF)
