# Supplementary material for: Spinel-Structured High Entropy Oxides: Low Temperature Synthesis, Characterization, and Potential Applications
Source: ACS Omega. 2025 Aug 25;10(35):39530–9. doi: 10.1021/acsomega.5c00902 (PMC12423803; doi:10.1021/acsomega.5c00902)
Supplement: Supplementary file 1 [file ao5c00902_si_001.pdf]

Spinel-Structured High Entropy Oxides: Low Temperature Synthesis, Characterization, and Potential Applications

Irem B. Algan Simsek,[1,2] Hussein O. Badr,[2,*] Neal Cardoza,[3] Erika Colin-Ulloa,[4] Gregory R. Schwenk,[2] Kaustubh Sudhakar,[2] Ulf Wiedwald,[5] Michael Farle,[5] Vibha Kalra,[6] Mohamed Ibrahim,[2] Lyubov V. Titova,[4] and Michel W. Barsoum[2, *]

[1]Department of Metallurgical and Materials Engineering, Gazi University, Ankara, 06560, Turkey

[2]Department of Materials Science and Engineering, Drexel University, Philadelphia, PA 19104, United States

[3]Department of Chemical and Biological Engineering, Drexel University, Philadelphia, PA 19104, United States

[4]Department of Physic, Worcester Polytechnic Institute, Worcester, MA | 01609 United States

[5]Faculty of Physics and Center for Nanointegration Duisburg-Essen, University of Duisburg-Essen, Duisburg, 47057, Germany

[6]Robert Frederick Smith School of Chemical and Biomolecular Engineering, Cornell University, Ithaca, NY 14853, United States

[*]Corresponding authors.

E-mail addresses: Badr, H.O. (hob28@drexel.edu), Barsoum, M.W. (barsoumw@drexel.edu).

Table S1. Summary of compositions, reagent type, molarity and synthesis conditions of HEOs

<table>
<tr><th>Composition</th><th>Aqueous Solutions (25 mL)</th><th>Solution Molarity</th><th>Synthesis Condition</th></tr>
<tr><td rowspan="5">FeNiCoCuZn</td><td>KOH</td><td>0.03</td><td>RT - 2h</td></tr>
<tr><td>KOH</td><td rowspan="3">1M for all bases except TMAH was 2.8M</td><td>RT – 2h
RT – 24 h
95°C- 24 h
95°C- 7 d</td></tr>
<tr><td>NaOH</td><td>95°C- 24 h</td></tr>
<tr><td>TMAH</td><td>95°C- 24 h</td></tr>
<tr><td>KOH</td><td>7</td><td>RT – 2h
95°C- 24 h</td></tr>
</table>

Table S2. The crystallite sizes of HEO-*colloid* are calculated with Scherrer formula

Sample	Average (nm)
1M KOH 95°C 24 h	12.3
1M KOH RT 24 h	4.2
1M NaOH 95°C 24 h	10.3
1M TMAH 95°C 24 h	11.2
Ref 3,4,6,22,24	from 12 - 18

Table S3. The OER activities of reported HEOs (in 1M KOH electrolyte)

**HEO Catalyst**	**Synthesis Method**	**Phase Structure**	**Average Particle Size (nm)**	**Overpotential (mV)**	**Ref**
FeCoNiCuZn-F	Hydrothermal reaction	spinel	180-250	290	6
FeNiCoCrMn	Solvothermal	spinel	2	407	16
FeCoNiMnZn	Hydrothermal	spinel	19	330	22
$FeCrCoNiAl_{0.1}$	DC reactive sputtering	spinel	Thin film	381	39
$FeNiCoCrMnS_2$	Solvothermal	bi-phased sulfides	unspecified	391	40
CoCrFeMnNi	Reverse co-precipitation	spinel	43-63	350	41
FeNiCo*CuZn*	Low temperature synthesis	spinel	12	460	This study

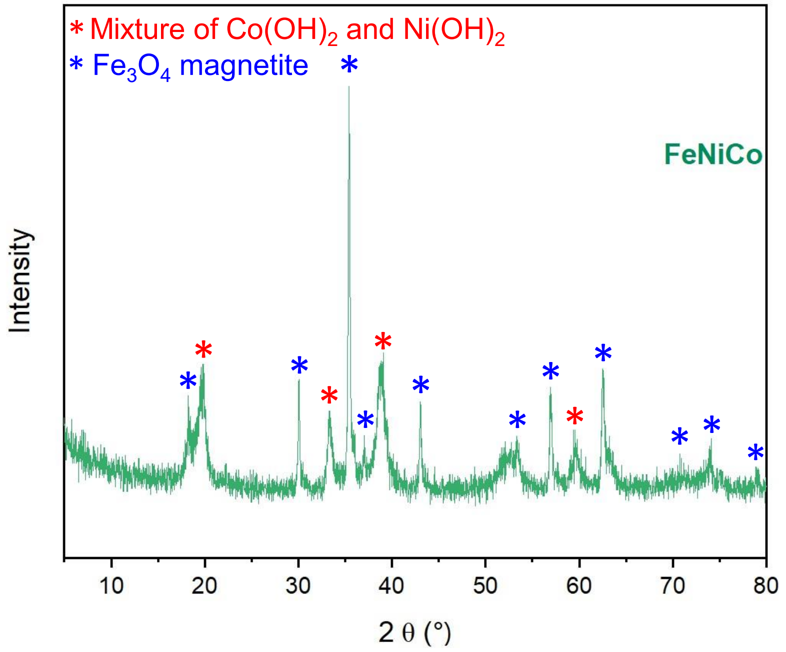

Figure S1. XRD pattern of a mixed metal oxides samples prepared by treating Fe, Ni, and Co salts (without adding Cu or Zn precursors) in 1M KOH solution at 95°C for 24h. Sample was processed similarly like the aforementioned details for HEO-*colloid*.

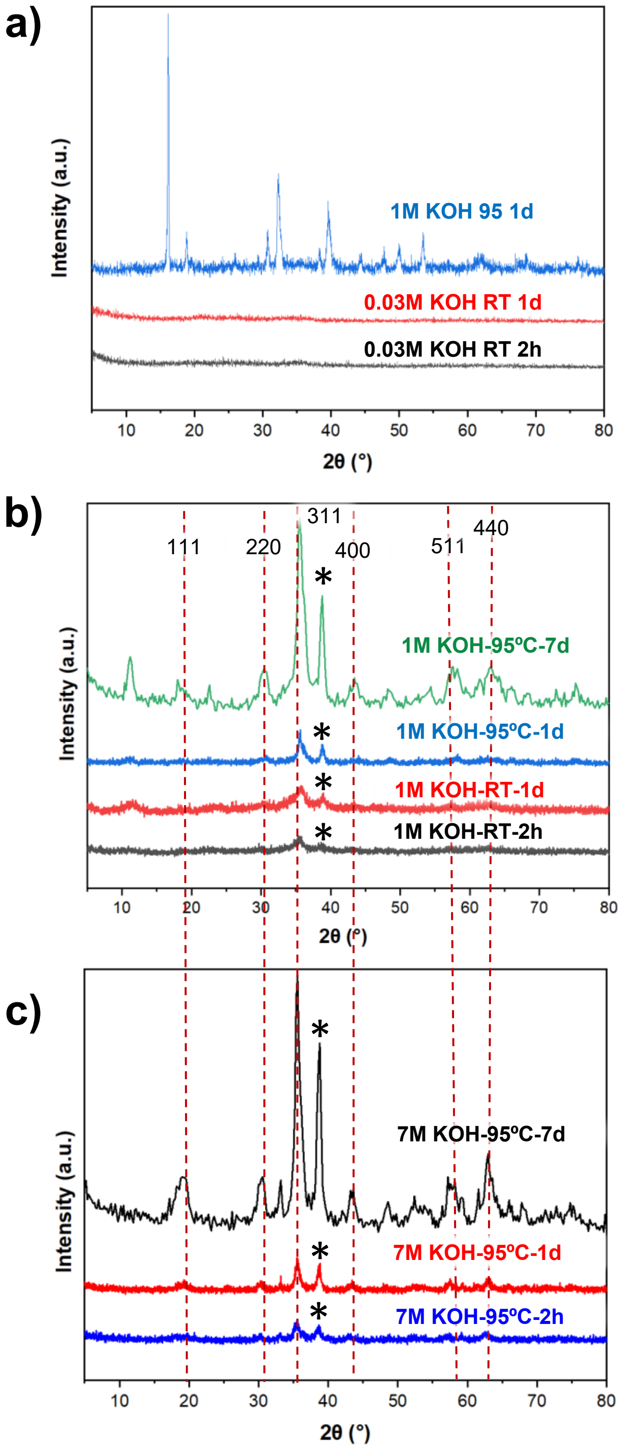

Figure S2. XRD patterns of HEO-*sediment* powders prepared by reacting Fe, Ni, Co, Cu, and Zn salts solutions with KOH solution following experimental conditions labeled on the panels. Powders were prepared using (a) 0.03M KOH at RT for 2h (black), 0.03 M KOH at RT for 1d (red), and 1M KOH at 95 °C for 1d (blue). (b) Powders were prepared using 1M KOH solution at RT for 2h (black), at RT for 1d (red), at 95°C for 1d (blue), at 95°C for 7d (green). (c) Powders ere prepared using 7M KOH solutions at 95 °C for 2h (blue), for 1d (red), and for 7d (black). Red vertical lines refer to 111, 220, 311,400, 511, and 440 planes in spinel-structured HEOs, respectively. Asterisks refer to CuO impurities in the resulting sediment. All samples were washed with ethanol and DI water 4-5 times then dried in open air at 50°C overnight.

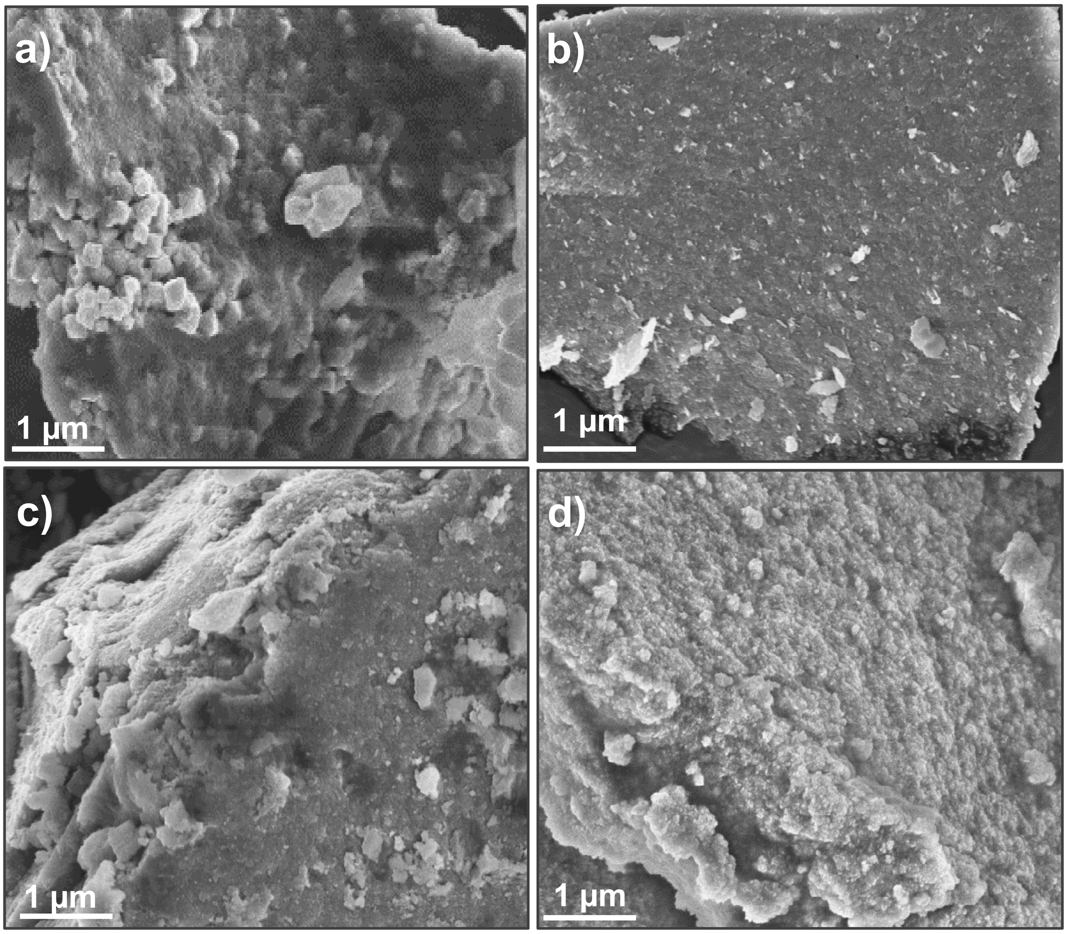

Figure S3: SEM micrographs of HEO-*colloid* reacted with (a) 2.8M TMAH at 95°C, (b) 1M KOH at RT, (c) 1M NaOH at 95°C, (d) 1M KOH at 95°C for 24h. All samples were washed with ethanol and DI water 4-5 times then dried in open air at 50°C overnight.

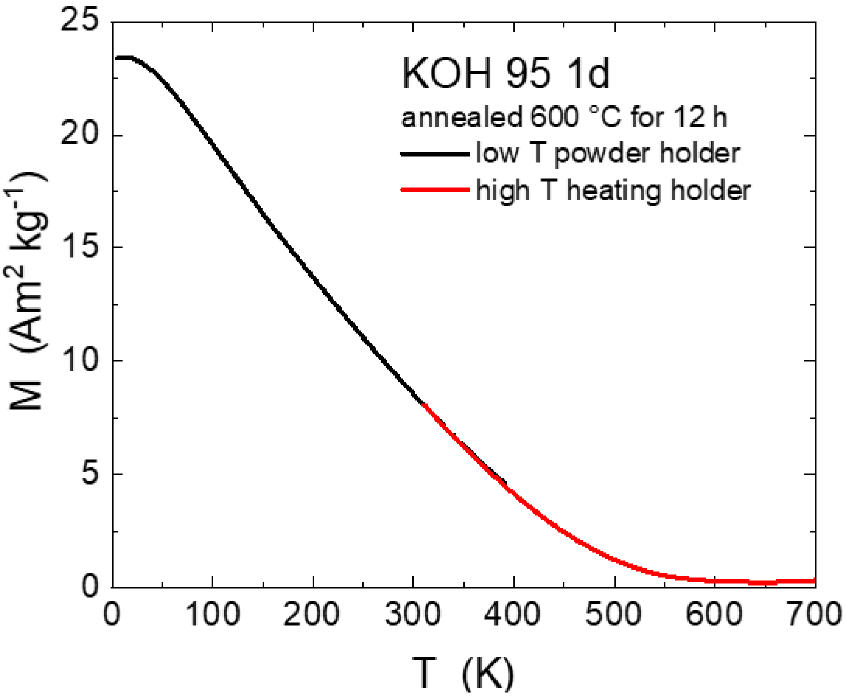

Figure S4: Temperature-dependent magnetization of HEO synthesized with 1M KOH at 95°C for 1d sample (black) in B = 1 T after additional annealing at T = 600 °C for 12 h (red). High temperature data was fit by a single factor to the magnetization data at low temperatures using the joint temperature interval of 300-390 K.

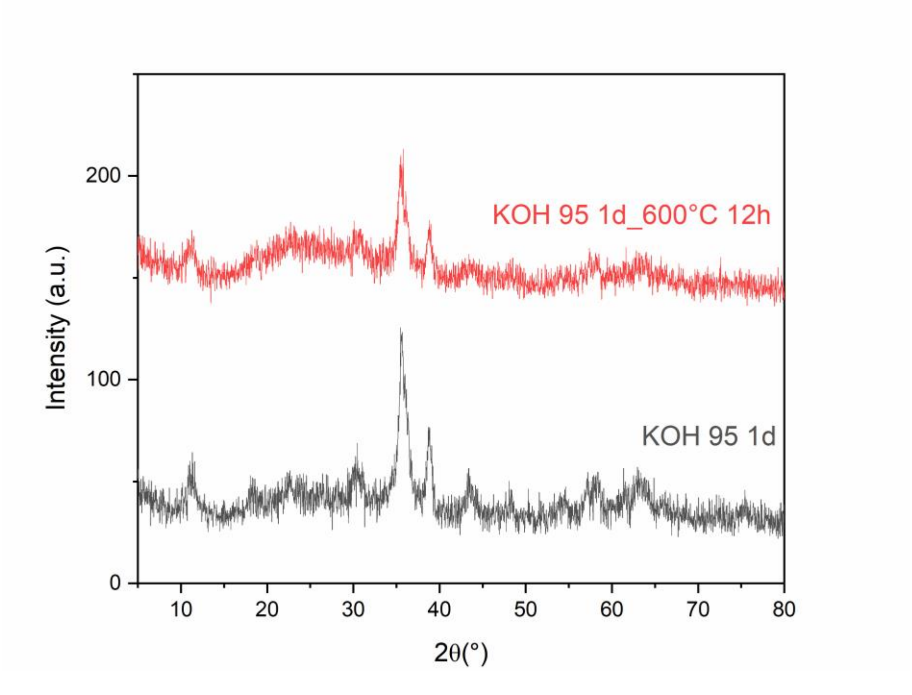

Figure S5: XRD of $(FeNiCoCuZn)_3O_4$ synthesized with 1M KOH at 95°C for 1d sample before (black) and after (red) annealing at T = 600 °C for 12 h
